# A Single Simulation Platform for Hybrid Photoacoustic and RF-Acoustic Computed Tomography

**DOI:** 10.3390/app8091568

**Published:** 2018-09-06

**Authors:** Christopher Fadden, Sri-Rajasekhar Kothapalli

**Affiliations:** 1 Department of Electrical Engineering, The Pennsylvania State University, University Park, PA 16802, USA; 2 Department of Biomedical Engineering, The Pennsylvania State University, University Park, PA 16802, USA; 3 Penn State Hershey Cancer Institute, The Pennsylvania State University, Hershey, PA 17033, USA

**Keywords:** photoacoustic imaging, tomography, thermoacoustic, radio frequency

## Abstract

In recent years, multimodal thermoacoustic imaging has demonstrated superior imaging quality compared to other emerging modalities. It provides functional and molecular information, arising due to electromagnetic absorption contrast, at ultrasonic resolution using inexpensive and non-ionizing imaging methods. The development of optical- as well as radio frequency (RF)-induced thermoacoustic imaging systems would benefit from reliable numerical simulations. To date, most numerical models use a combination of different software in order to model the hybrid thermoacoustic phenomenon. Here, we demonstrate the use of a single open source finite element software platform (ONELAB) for photo- and RF-acoustic computed tomography. The solutions of the optical diffusion equation, frequency domain Maxwell’s equations, and time-domain wave equation are used to solve the optical, electromagnetic, and acoustic propagation problems, respectively, in ONELAB. The results on a test homogeneous phantom and an approximate breast phantom confirm that ONELAB is a very effective software for both photo- and RF-acoustic simulations, and invaluable for developing new reconstruction algorithms and hardware systems.

## 1. Introduction

One of the goals of modern medical imaging is to simultaneously provide molecular, functional, and structural/anatomical information corresponding to various tissues. To achieve such comprehensive information, a combination of conventional imaging technologies such as X-ray computed tomography (CT) [1], positron emission tomography (PET) [2] and magnetic resonance imaging (MRI) [3] are used. CT provides anatomical contrast, PET provides molecular and metabolic information, and MRI provides both functional and anatomical contrasts. A PET–CT or PET–MRI combination is therefore widely used for simultaneously mapping molecular and anatomical contrasts [4–6].

All of these methods have significant downsides: one of which is the financial burden, which means that these methods are not ideal for routine imaging, and a second is the ionizing radiation used in CT and PET. As an alternative, ultrasound imaging that uses non-ionizing radiation is routinely used in several clinical applications for anatomical imaging [7]. However, it lacks the molecular or functional information necessary for detecting the early symptoms of disease.

Alternative medical imaging modalities that provide anatomical, functional, and molecular information about the tissue, while being lower cost and not being as restrictive to patient movement, are needed. Photoacoustic computed tomography (PACT) and radio frequency (RF)-induced acoustic computed tomography (RACT), together known under the general term thermoacoustic computed tomography (TACT), match these objectives of lower cost functional/molecular imaging, using non-ionizing electromagnetic radiation [8–16]. While PACT maps optical absorption contrast using optical radiation induced acoustic wave detection, RACT maps tissue conductivity using RF induced acoustic wave detection. More importantly, since these hybrid (combining electromagnetic radiation and acoustic detection) imaging modalities share the same ultrasound detection platform, combined trimodality PACT–RACT–UCT (ultrasound computed tomography) systems have been realized for mapping functional, molecular, and anatomical contrasts [17]. The molecular absorption of electromagnetic energy causes thermal expansion in the tissue, which then leads to generation of acoustic waves. The acoustic waves propagate out of the tissue and are received by ultrasound transducers located at the boundary of the body. This data is then used to reconstruct thermoacoustic images displaying electromagnetic absorption contrast at ultrasonic spatial resolution. The imaging depth and spatial resolution in TACT is scalable with the frequency of excitation radiation and ultrasound transducer. The fact that the detected acoustic signal arises directly from specific molecules inside the tissue makes TACT a molecular/functional imaging technology. In PACT, the tissue chromophores—such as oxy-hemoglobin, deoxy-hemoglobin, melanin, and lipids—absorb light photons in the wavelength range from 400 nm to 1200 nm. By using different wavelengths, which takes advantage of the resonance peaks in the absorption spectrum of the imaged molecules, the distribution of different molecules inside the tissue can be mapped. In RACT, a radio frequency source in the frequency range from 434 MHz to 9 GHz is used for mapping the water distribution. The thermoacoustic effect in this frequency range is dependent on the conductivity distribution of the medium. The conductivity difference between water, tissue, and tumors can then give useful functional images.

Accurate numerical modeling of TACT is paramount for the development of robust reconstruction algorithms to quantify the electromagnetic absorption properties of the tissue. In PACT, the forward optical simulation of total light fluence (Φ), calculated inside the tissue medium, is usually achieved using either Monte Carlo simulations or the software package NIRFast, which solves the light diffusion equation. The fluence distribution is then converted to an initial pressure rise, which is further propagated through the tissue medium and detected by the ultrasound transducers located on the boundary using acoustic simulation tools such as the K-wave toolbox. NIRFast [18] uses the finite element method (FEM), while K-wave uses a spectral-based finite difference method (FDM) to model the propagation of acoustic waves [19]. The goal of the reconstruction problem is then to recover the tissue properties (optical absorption in PACT and conductivity in RACT) given only the sensor data.

Groups that use a single simulation platform for both the optical and acoustic propagation needed for the hybrid PACT/RACT technique are less common. Recently, there are studies that use the commercial software COMSOL to solve both propagation problems with a single software package [20,21]. As an alternative to the commercial software, the simulation system described in this paper uses the open source softwares Gmsh [22] and GetDP [23], often combined under the name ONELAB [24]. ONELAB is an FEM solver, which uses Gmsh for creating the FEM mesh, and GetDP for solving generic partial differential equations (PDEs) with the FEM method. Advantages of using Gmsh include its ability to create user defined meshes, but also having standard interfaces with other commonly used mesh and computer-aided design (CAD) software such as STEP, IGES, and STL. Segmented DICOM images commonly used in MRI can then be converted to a mesh that Gmsh understands, creating realistic phantoms on which to test algorithms. The ability of GetDP to solve generic PDEs allows the user to implement algorithms to solve for optical and acoustic propagation, similar to the combination of NIRFast and K-wave. Both propagation methods, as well as any reconstruction methods, are implemented in the same mesh, with no loss of precision. Since there is less chance of numerical error, a single software platform represents a more accurate approximation of a real-world scenario. Besides PACT with optical sources, we demonstrate that the generality of GetDP allows for sources in the RF regime of the electromagnetic spectrum to also be simulated for RACT. The use of the ONELAB software package allows accurate TACT modeling, in order to develop algorithms for functional imaging of the human body. Although there are experimental studies combining PACT and RACT techniques into one setup, perhaps our study is the first to report a single TACT simulation platform for simulating both PACT and RACT.

The rest of the paper is organized as follows: In Section 2, we describe methods and materials. Sections 3.1 and 3.2 show PACT and RACT results on a homogeneous phantom. Sections 3.3 and 3.4 show PACT and RACT results on an approximate breast phantom. Section 4 is a discussion of the results in Section 3, comparing errors in reconstruction between PACT and RACT, and between the two phantoms. Section 5 concludes the paper.

## 2. Materials and Methods

**Figure 1.**
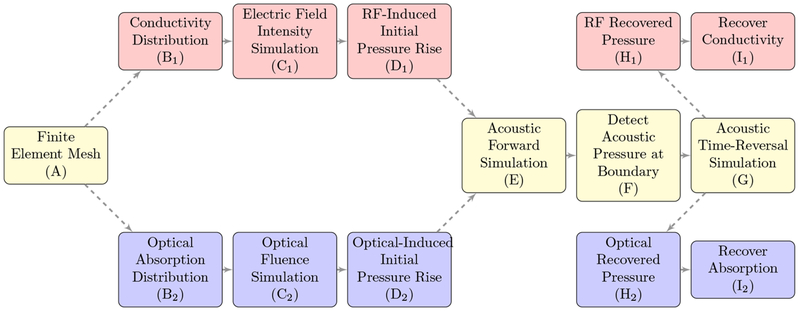
The similarities between key steps of the photoacoustic computed tomography (PACT, blue) and radio frequency (RF)-induced acoustic computed tomography (RACT, red) workflows, with methods that are shared represented in yellow. Step A represents the finite element mesh in Section 2.5, used for both types of simulation. Step B_1_ is represented in Figure 9a, while step B_2_ is represented in Figure 7a. Step C_2_ uses the optical diffusion Equation (1). Step C_1_ uses the electromagnetic wave Equation (5). The resulting pressure rise for the optical (D_2_) and RF (D_1_) radiations are represented by Figures 7b and 9b, respectively. The initial pressure propagates out to the boundary (E) via the scalar wave Equation (7). The data received by acoustic sensors at the boundary (F) can then be used in reconstruction algorithms (G) to recover the initial pressure. In this paper, a time-reversal algorithm is applied for reconstruction (Section 2.4). The reconstructed pressure is then divided by the intensity (I_2_, I_1_) to recover the recover the absorption or conductivity for the optical and RF cases, shown in Figures 7c and 9c, respectively.

There are three main phenomena that need to be modeled in thermoacoustics. The first is the propagation of the initial energy source, in this study near infrared optical or radio frequency electromagnetic waves, and calculation of the total fluence/intensity distribution inside the tissue medium. The second phenomenon is the acoustic propagation, after calculating an initial pressure from the intensity distribution. The third phenomenon is the reconstruction of the tissue parameters, which depend on the type of energy source used for tissue excitation. A flowchart showing the similar steps between RACT and PACT is shown in Figure 1. This study is focused on using a time reversal [25,26] reconstruction method to recover the initial pressure, followed by a simple division to find the tissue parameters. There are several alternative approaches for reconstructing the tissue parameters that can be integrated with our study in the future. Back-projection methods may be used when using specific geometries [27] and parametrix methods for more general geometries [28]. The back-projection methods are analytically exact, but only for geometries with a special symmetry, and with a constant speed of sound. Parametrix methods do not provide analytically exact methods, but instead give approximations with error bounds for general geometries, and can even be developed for regions with varying speeds of sound. A survey of several reconstruction methods is given in Reference [29].

### 2.1. Optical Propagation for Photoacoustic Computed Tomography (PACT)

A full description of optical propagation would use the radiative transport equation in its full generality. This equation can be modeled by the use of Monte Carlo methods, with significant computational cost [30,31]. Since the the photoacoustic effect depends on the optical fluence rate, and the wavelengths used correspond to the near infrared (NIR) regime, a diffusion approximation to the radiative transport equation is often used [32]. The diffusion equation for the fluence rate is:

(1) $$-\nabla \cdot \kappa (x)\nabla \Phi (x,\lambda )+\mu_{a}(x)\Phi (x)=q(x)\;x\in \Omega$$

(2) Φ(x)=0x∈∂Ω.

In this equation, Φ is the optical fluence rate, or intensity of the light. *μ_a_* is the absorption coefficient at each point in space, while $\kappa =\frac{1}{3(\mu_{a}+\mu_{s}^{\prime })}$ is the diffusion coefficient, calculated using the absorption coefficient and the reduced scattering coefficient $\mu_{s}^{\prime }$. *q* is the source function, the initial laser pulse that irradiates the tissue medium. Ω is the tissue domain of interest, usually a subset of $R^{n}$, with *n* corresponding to the dimension of the problem (2 or 3). Equation (2) corresponds to the Dirichlet boundary condition used in this study The amount of pressure generated by the optical energy is given by a constant of proportionality, the Gruneisen parameter $\Gamma =\frac{\alpha v_{s}^{2}}{C_{p}}$, where *α* is the volume thermal expansion coefficient, *v_s_* is the speed of sound, and *C_p_* is the heat capacity at constant pressure [33]. Therefore, for a given fluence rate Φ, the initial pressure is given by:

(3) $$p_{0}(x)=\Gamma (x)\Phi (x)\mu_{a}(x).$$

While Γ will physically vary over space, this small deviation is ignored in this study, and the parameter is assumed constant, with a value of 0.1, which approximates standard tissue.

### 2.2. Radio Frequency Propagation for RF Acoustic Computed Tomography (RACT)

Radio frequency radiation is modeled by Maxwell’s equations, which when modeled at a single frequency can be reduced to the following wave equation:

(4) $$\nabla \times \nabla \times E(x)-\omega^{2}\mu (x)\varepsilon (x)E(x)=q(x)\;x\in \Omega .$$

In this equation, *E* is the complex electric field, *ω* = 2*πf* is the frequency, and *μ* and ε are the permeability and permittivity of the medium, respectively. In a material with electric loss, the permittivity is complex valued, with $\varepsilon =\varepsilon_{\text{real}}-j\frac{\sigma }{\omega }$, and *σ* = 0 for a medium with no loss. When the simulation is restricted to two dimensions (assuming a *TM_z_* polarization), Equation (4) can be reduced to a scalar Helmholtz equation:

(5) $$\nabla^{2}E_{3}(x)+k{\left(x\right)}^{2}E_{3}(x)=q(x)\;x\in \Omega ,$$

where $k=\frac{\omega }{c}$ is the wavenumber, and can be complex in a tissue with energy loss. For boundary conditions, a Dirichlet boundary condition similar to Equation (2) is implemented. However, this would not prevent the electromagnetic waves from reflecting off the boundaries of the domain, causing numerical artifacts. Therefore, while there is a Dirichlet boundary condition, there is also a non-physical space occupied by a perfectly matched layer (PML) to absorb the outgoing waves and prevent reflection [34]. Once the electric field is calculated, the initial pressure is given by three contributions [35]. The first contribution is due to the conductivity of the medium, $p_{\mathrm{cond}}=\int_{V}\frac{\sigma }{2}{|E|}^{2}\mathrm{dV}$. The second due to the permittivity $\int_{V}\frac{\varepsilon_{0}\varepsilon_{r}}{2}{|E|}^{2}\mathrm{dV}$, and the third the permeability $\int_{V}\frac{\mu_{0}\mu_{r}}{2}{|H|}^{2}\mathrm{dV}$. In practice, for the tissue media of interest in thermoacoustic tomography, the contribution to the pressure due to the conductivity dominates over the permittivity and permeability, and so these terms are generally ignored. Therefore, similar to the optical absorption in Equation (3), the initial pressure due to the electric field is given as:

(6) $$p_{0}(x)=\Gamma (x)\frac{\sigma (x)}{2}{|E(x)|}^{2}.$$

### 2.3. Thermoacoustic Equation

Once an initial pressure is found, via either optical or radio frequency electromagnetic radiation, the pressure must then propagate to ultrasound transducers located outside the tissue boundary. Extremely short (nanosecond) pulses of energy are assumed to be irradiating the medium. Therefore the initial pressures are assumed to be delta functions, and only present for the initial conditions when formulating the equations. The pressure wave in photoacoustics is usually modelled using a standard scalar wave equation, with initial conditions:

(7) $$\frac{\partial^{2}}{\partial t^{2}}p(x,t)=c^{2}(x)\nabla^{2}p(x,t)\;x\in \Omega \;p(x,0)=f(x)\;\frac{\partial }{\partial t}p(x,0)=0,$$

where, *f*(*x*) corresponds to the initial pressure found from the initial pulsed excitation, and *p*(*x*, *t*) is the pressure wave that propagates to the transducers. Similar to the electromagnetic wave equation, the pressure propagation also requires a perfectly matched layer (PML) to prevent reflections from the boundary of the numerical domain [36,37]. Therefore, the pressure is received at transducers along a curve (surface in 3D) *γ*, which is not the boundary of the domain ∂Ω.

### 2.4. Time Reversal Reconstruction Algorithm

The pressure received at the transducers can be represented as *g*(*y*, *t*), with *y*∈*γ*. The goal of the reconstruction problem is to reconstruct the tissue parameters, *μ_a_* in the optical excitation or *σ* in the RF excitation case, given *g*(*y*, *t*). One way of recovering the parameters is to try and find the initial pressure *f*(*x*) from the pressure measurements *g*(*y*, *t*), and then divide the pressure by the assumed known energy distribution to recover the parameters. One of the more common methods to recover the initial pressure is using the time-reversal technique. For the time-reversal to rigorously reconstruct *f*(*x*), requires Huygen’s principle to be valid. Unfortunately, Huygen’s principle does not hold when the speed of sound is not constant, and more problematically, it does not hold in two dimensions [38]. Therefore, in most domains of interest the time-reversal method recovers an approximation of the initial pressure *f*(*x*).

As the name suggests, time reversal entails taking the received pressure *g*(*y*, *t*), and using it as a source on *γ*, with time moving in reverse. If the forward simulation was run until a stoppage time *T*, then when simulated using the time reversal method *p*(*x*, *T*) ≈ *f*(*x*). There are several other methods that can be used to recover *f*(*x*) besides time-reversal, but there is no universally accepted reconstruction algorithm that works in all cases of interest. Most of these methods require pressure information at the sensors as functions of time. For this reason, the computationally expensive time-domain wave equation was used for solving the pressure wave equation instead of a Helmholtz equation similar to Equation (5). Since the initial pressure acts as a delta function, the received signal is inherently broadband. A time domain simulation is more efficient for measuring broadband response, and provides a better representation of what would be measured during a physical experiment.

### 2.5. Phantom Geometry

Two phantoms are used to demonstrate the effectiveness of the single simulation tool used in this study. The first homogeneous phantom consists of a circular region of interest with radius 20 mm. Within this region, two objects are placed, a circle of radius 1 mm placed approximately 4 mm to the left of center, and an ellipse with major axis 2 mm and minor axis 1 mm placed 4 mm to the right of center. The second phantom consists of an approximation to a human breast. The breast region of interest is represented by a circle of radius 50 mm. Glandular tissue rendered as a circle of radius 10 mm surrounds an elliptical tumor with major axis 7 mm and minor axis 3.5 mm. The tumor is located 32.5 mm deep from the right side of the phantom. The optical sources and acoustic detectors are placed around the region of interest in a continuous fashion.

For creating the finite element meshes, a characteristic length of 2 mm was used for efficiency reasons, though near the absorbing objects, the size of the elements smoothly decreased in order to properly model the objects with sufficient resolution. For the homogeneous phantom mesh, the total number of nodes in the mesh was 3635 with 7268 elements. The mesh for the breast phantom had 12,166 nodes and 24,330 elements. For the time domain simulation of the acoustic propagation, a time step of 30 nanoseconds was used in a Newmark numerical integration method. The homogeneous phantom ran for 700 time steps, while the breast phantom ran for 1700 time steps. A constant speed of sound of 1.5 mm/μs was used for both the forward and time-reversed simulations.

The time estimates for the breast phantom are: 0.6 s for the optical simulation, and 1.1 s for the RF simulation. The acoustic simulation took 18.1 s in the optical source case, and 17.3 s in the RF source case. The reconstruction using time reversal took 385.8 s and 408.0 s for the optical and RF source cases, respectively. The homogeneous phantom timings are: 0.16 s and 0.315 s for the optical and RF simulations; 2.1 s and 2.0 s for the forward acoustic simulation; and the time reversal took 38.4 s and 39.4 s for the optical and RF cases, respectively. The timings were done on a Dell Precision 5820 desktop PC, on a single thread. ONELAB has the functionality to run on multiple threads, as well as a graphical processing unit (GPU), but these options were not used in measuring these time estimates.

### 2.6. Phantom Parameters

The absorption coefficient *μ_a_* of the homogeneous phantom for the background was set to 0.001 mm^−1^. The circle and ellipse, which represent absorbers, have an absorption coefficient of 0.425 mm^−1^; the average absorption coefficient of blood at 800 nm. As is common in human tissue, the background is assumed to have a higher reduced scattering coefficient $\mu_{s}^{\prime }$ than the absorbers. The reduced scattering coefficient for the background is set to 1 mm^−1^, while the absorbers $\mu_{s}^{\prime }$ is set to zero for this phantom. In terms of electrical properties, the human body in general does not have a significant magnetic response at radio frequencies, and so the relative permeability *μ_r_* is set to 1 for all objects. A background relative permittivity of ε_*r*_ = 5 is similar to human tissue at 434 MHz. Unlike in the PACT case, objects such as tumors have a higher scattering coefficient as well as absorption in the radio frequency regime. Therefore, the relative permittivity of the circle and ellipse were set to ε_*r*_ = 25. The conductivity, which governs the amount of absorption, was set to 0.1 S/m for the background, approximating general tissue. The circle and ellipse are given values similar to that of a tumor, 10 S/m [39].

General breast tissue has an absorption coefficient *μ_a_* = 0.005 mm^−1^, with a reduced scattering coefficient of 1.52 mm^−1^ when an 800 nm source is used. The optical properties of the glandular tissue and tumor depend on the assumed material composition of the tissues. The assumed amount of hemoglobin and percentage of water can have a dramatic effect on the properties at any given wavelength. In this study, the same composition as Reference [18] is used for the glandular and tumor tissue. The spectral characteristics of hemoglobin, deoxyhemoglobin, and water at 800 nm are taken from References [40,41]. The specific optical properties used for this breast phantom are given in Table 1.

Human tissue is dispersive at radio frequencies, and so the electrical properties vary over frequency, though not as dramatically as the optical parameters. The properties for the generic and glandular tissue at 434 MHz were taken from the ITIS database provided by ETH-Zurich [42], which references a technical report compiled by the United States Air Force [43,44]. The tumor properties were extrapolated from data provided at 100 MHz in Reference [39] using a Cole–Cole dispersion model. The specific electromagnetic parameters are given in Table 2. The relative magnetic permeability is again set to unity, since the body does not exhibit strong magnetic response at 434 MHz.

## 3. Results

The ability of the ONELAB software platform to simulate both PACT (optical source) and RACT (radio frequency source) with the same tool and on the same mesh, is demonstrated using the phantoms described in the methods section. Below we first present PACT and RACT results for the homogeneous phantom embedded with two absorbers and then the results for the breast phantom with tumor.

### 3.1. Photoacoustic Computed Tomography (PACT) of the Homogeneous Phantom

**Figure 2.**
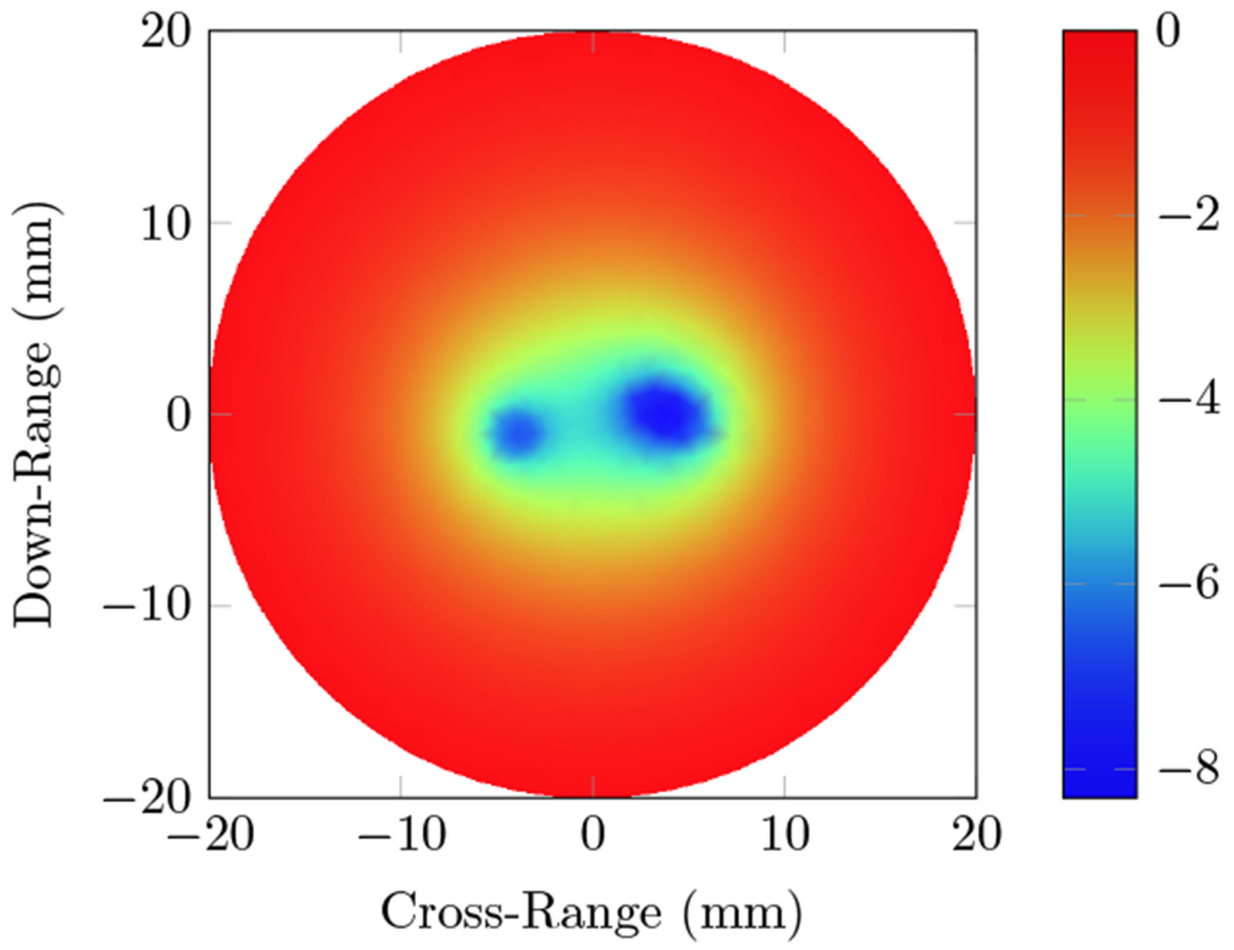
Total optical fluence distribution inside the homogeneous phantom with two absorbers using an 800 nm source. The circular and elliptical absorbers, simulating hemoglobin with absorption coefficient *μ_a_* = 0.425 mm^−1^ at 800 nm, are located 4 mm to the left and right of the center. The background has a *μ_a_* = 0.001 mm^−1^, and a reduced scattering coefficient $\mu_{s}^{\prime }=1$ mm^−1^. $\mu_{s}^{\prime }=0$ for the absorbers.

The optical excitation for the first phantom uses a wavelength of 800 nm. This specific wavelength was chosen as it is often used in photoacoustics, due to the absorption spectrum of the absorbers of interest, such as hemoglobin. With the homogeneous phantom parameters, the simulated optical fluence is given in Figure 2.

**Figure 3.**
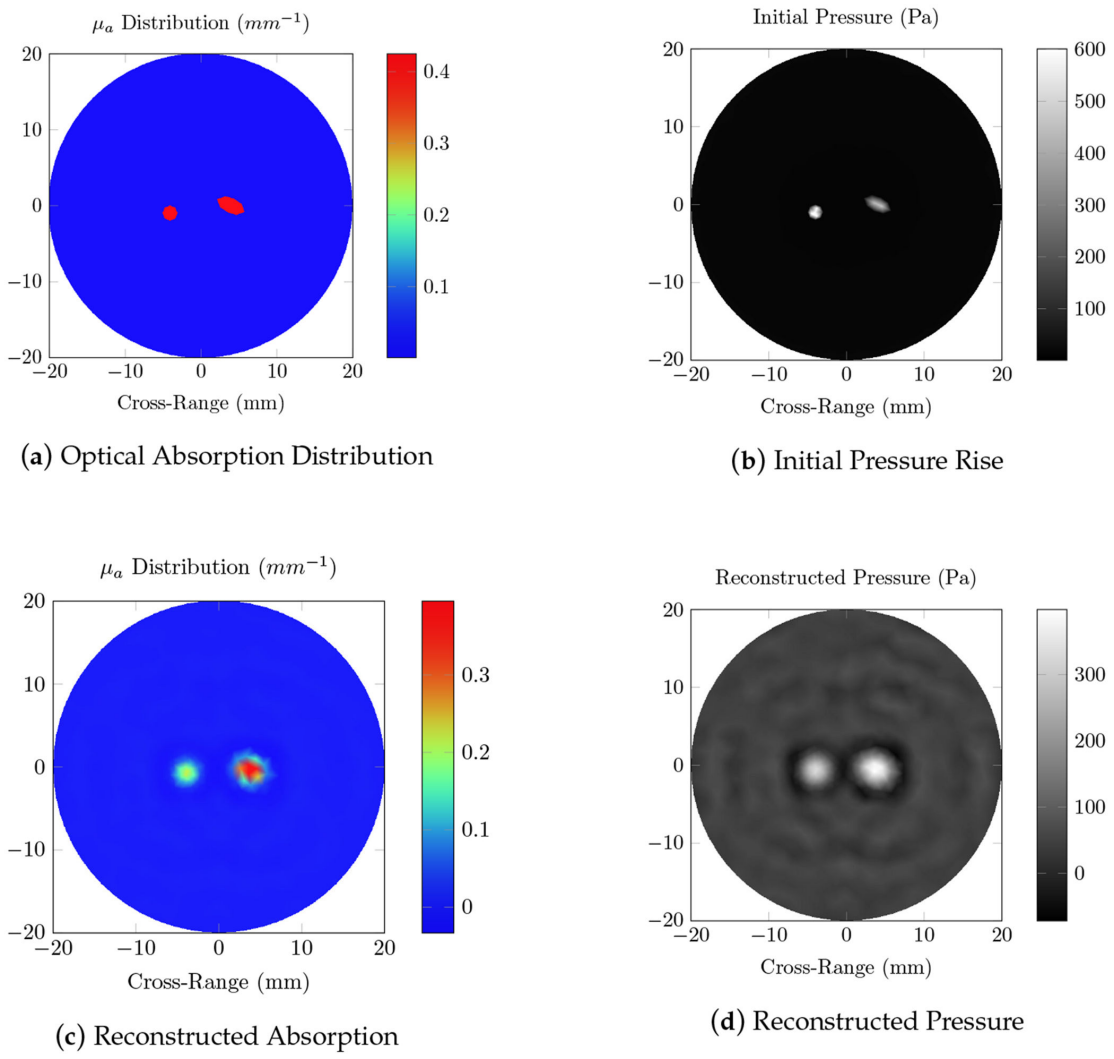
Photoacoustic computed tomography (PACT) simulations using an 800 nm source on the homogeneous phantom with two light absorbing inclusions; (**a**) the true optical absorption distribution; (**b**) the initial pressure rise induced by the photoacoustic effect; (**d**) the reconstructed pressure, calculated using the time-reversal algorithm; and (**c**) the reconstructed absorption, obtained by dividing the reconstructed pressure with the fluence.

The position of the absorbers is easily approximated by the nulls in the fluence distribution. The approximate shapes can be identified, which can be used for more stable reconstruction of the material parameters. The 8 mm spacing is large enough to identify two distinct absorbers, with the ultimate resolution governed by the reconstruction of the fluence that would be done in practice. The initial pressure induced by this fluence distribution, as well as the original and reconstructed absorption coefficient images are provided in Figure 3.

The fluence is not constant across the domain, and so the two absorbers induce slightly different initial pressures. The larger object is reconstructed with less error, since it is approximately constant over a larger area. Non-idealities in the reconstructed background pressure are suppressed by using the fluence, and so the reconstructed objects are clearly separated from the background.

### 3.2. RF-Acoustic Computed Tomography (RACT) of the Homogeneous Phantom

**Figure 4.**
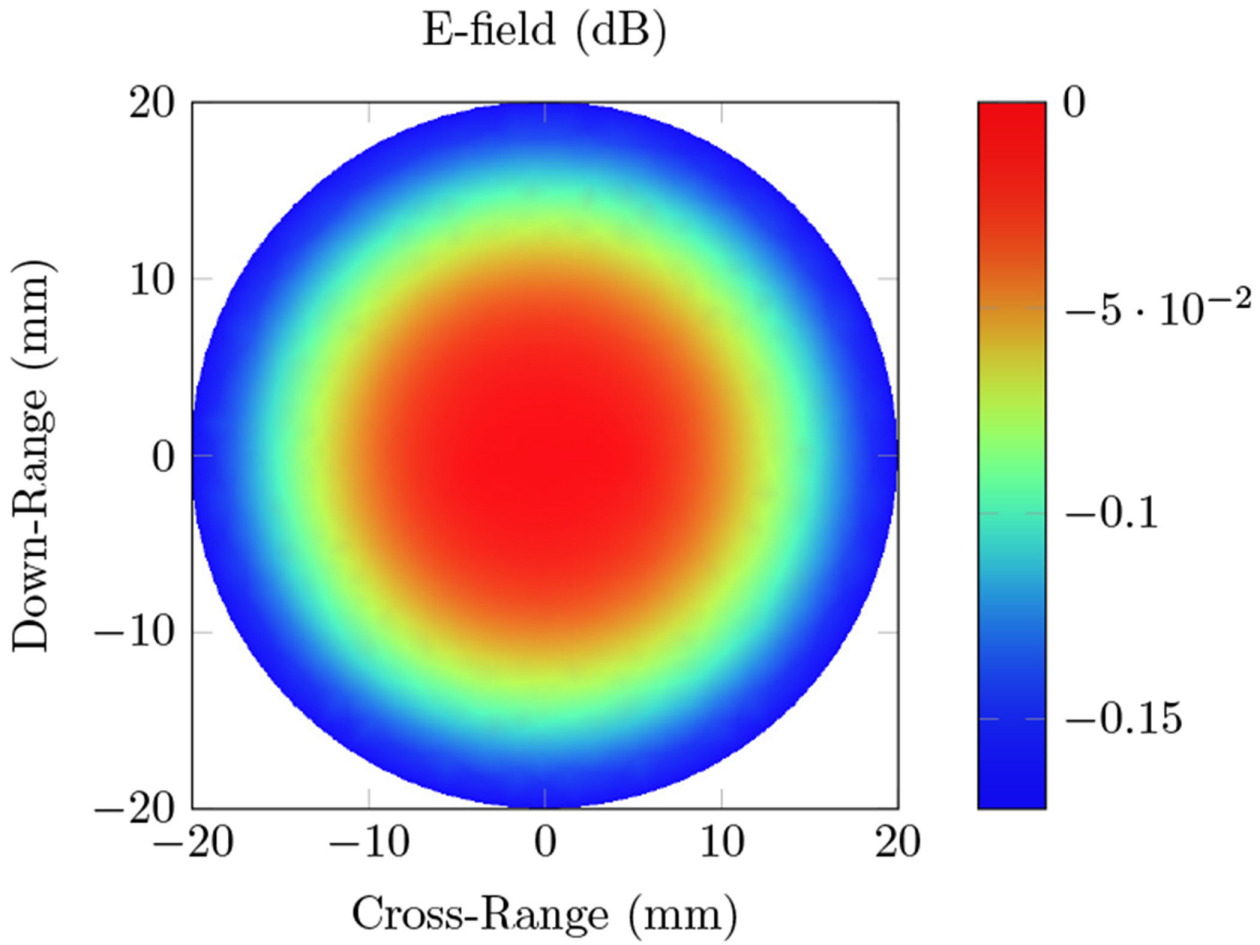
Total electric field intensity distribution inside the homogeneous phantom containing two absorbers, using a 434 MHz radio frequency (RF) source. The circular and elliptical absorbers, with conductivity *σ* = 10 S/m, are located 4 mm to the left and right of the center, and have a dielectric constant ε_*r*_ = 25. The background has *σ* = 0.1 S/m, and ε_*r*_ = 5.

The RACT simulation used the same finite element mesh that was used for the photoacoustic simulation, utilizing a typically used radio frequency source of 434 MHz. For the parameters given in the methods section, the electric field magnitude is provided in Figure 4.

**Figure 5.**
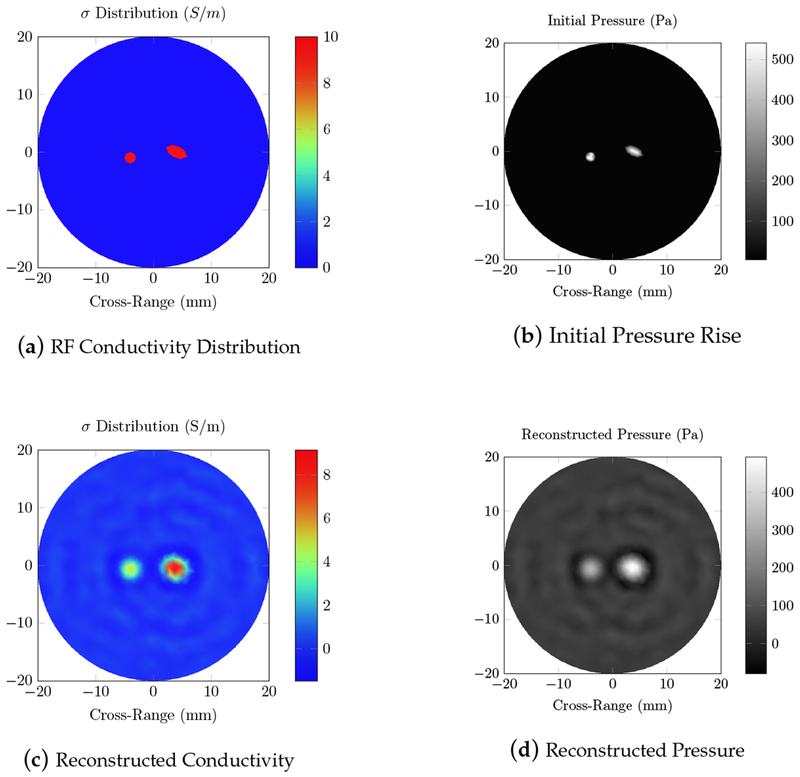
Radio frequency (RF)-induced acoustic computed tomography (RACT) simulations using a 434 MHz RF source on the homogeneous phantom with two absorbers. (**a**) The true RF conductivity distribution; (**b**) the initial pressure rise induced by the thermoacoustic effect; (**d**) the reconstructed pressure, calculated using the time-reversal algorithm; and (**c**) the reconstructed conductivity, obtained by dividing the reconstructed pressure by the electric field intensity.

Unlike the PACT case, the electric field alone does not provide any information on the location of the absorbers. Since the wavelength is much larger than the simulation domain, and there is small difference in conductivity between the absorbers and background, the electric field intensity is approximately constant. The results of the RF acoustic simulation of this homogeneous phantom are shown in Figure 5.

### 3.3. Photoacoustic Computed Tomography (PACT) of the Breast Phantom

**Figure 6.**
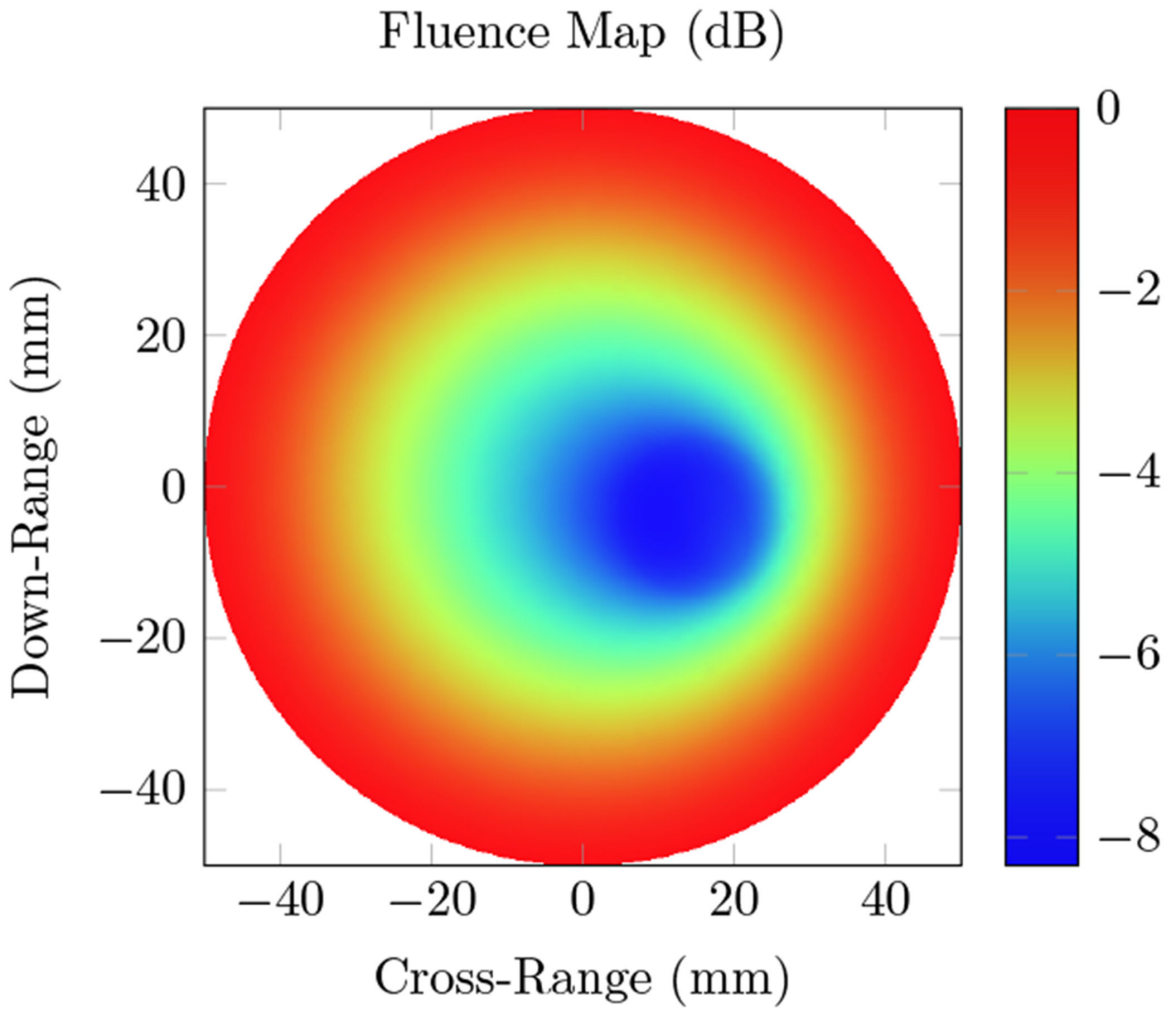
Total optical fluence distribution using an 800 nm wavelength source on the breast phantom. The background absorption coefficient is *μ_a_* = 0.0005 mm^−1^, with a reduced scattering coefficient $\mu_{s}^{\prime }=1.5742$. Glandular tissue (*μ_a_* = 0.0059 mm^−1^, $\mu_{s}^{\prime }=1.12\;{\mathrm{mm}}^{-1}$) surrounds a tumor (*μ_a_* = 0.0021, $\mu_{s}^{\prime }=0.625\;{\mathrm{mm}}^{-1}$) located 32.5 mm deep from the right side of the phantom.

**Figure 7.**
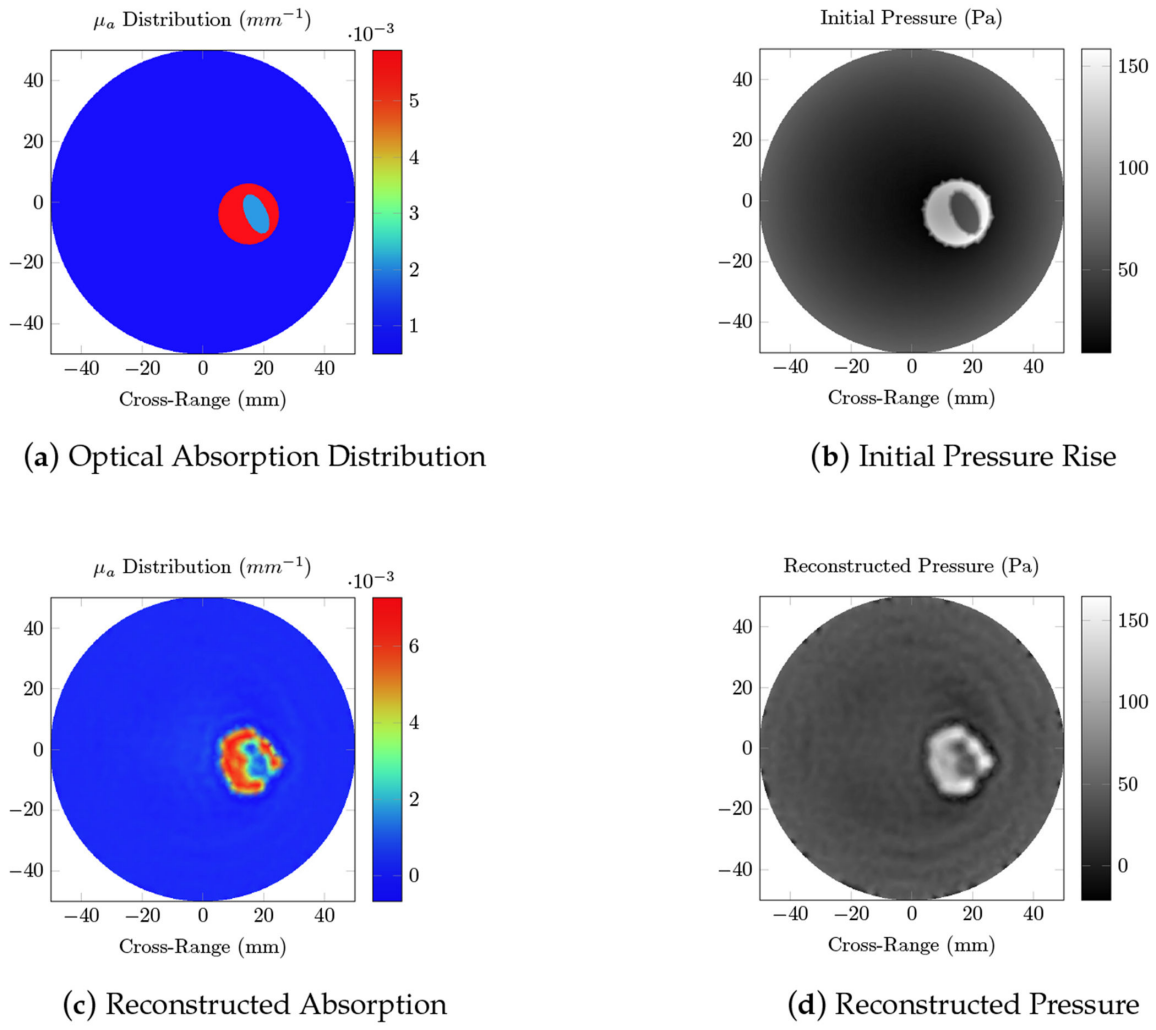
Photoacoustic computed tomography (PACT) simulations of the breast phantom at 800 nm wavelength; (**a**) the true optical absorption; (**b**) the initial pressure rise induced by the photoacoustic effect;(**d**) the reconstructed pressure, calculated using the time reversal algorithm; and (**c**) the reconstructed absorption, obtained by dividing the reconstructed pressure with the fluence. The elliptical region similar to the background is the tumor, surrounded by the glandular tissue.

Figure 6 shows the fluence distribution generated by ONELAB for the breast phantom described in the methods section. A rough estimate of the tumor location can be predicted from the fluence map, but no shape information can be obtained from the fluence distribution alone. The photoacoustic simulation is able to identify the shape of the tumor, as well as the difference between cancerous and glandular tissue. Results in Figure 7 show the initial pressure rise and the resulting reconstructed absorption distribution.

### 3.4. RF-Induced Acoustic Computed Tomography (RACT) of the Breast Phantom

**Figure 8.**
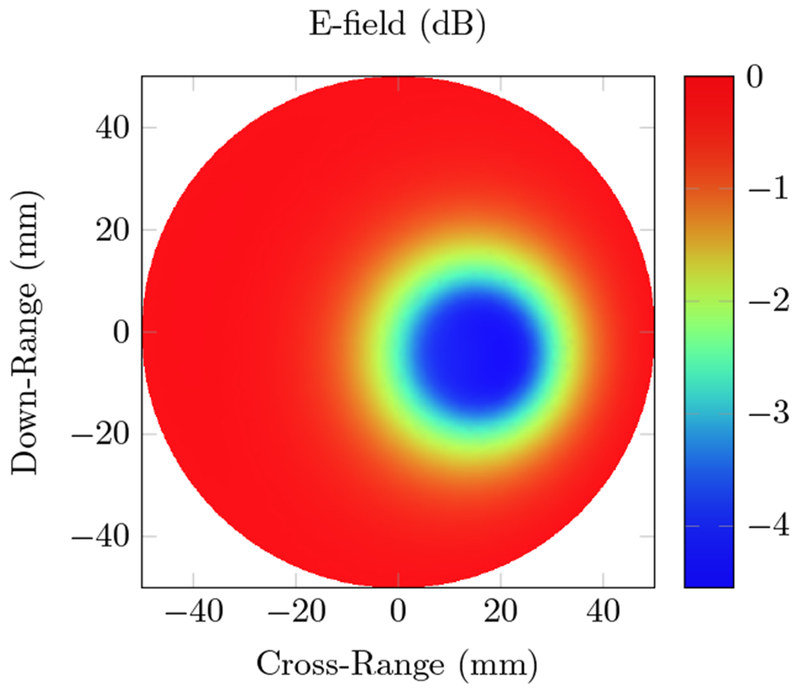
Total electric field intensity distribution of a 434 MHz radio frequency source inside the breast phantom. The background has a conductivity *σ* = 0.0353 S/m and dielectric constant ε_*r*_ = 5.51. Glandular tissue (*σ* = 8.86 S/m, ε_*r*_ = 61.3) surrounds a tumor (*σ* = 13.03 S/m, ε_*r*_ = 25.25) located 32.5 mm deep from the right side of the phantom.

The radio frequency source for the breast phantom, similar to the homogeneous phantom, operates at 434 MHz. The field distribution is provided in Figure 8.

**Figure 9.**
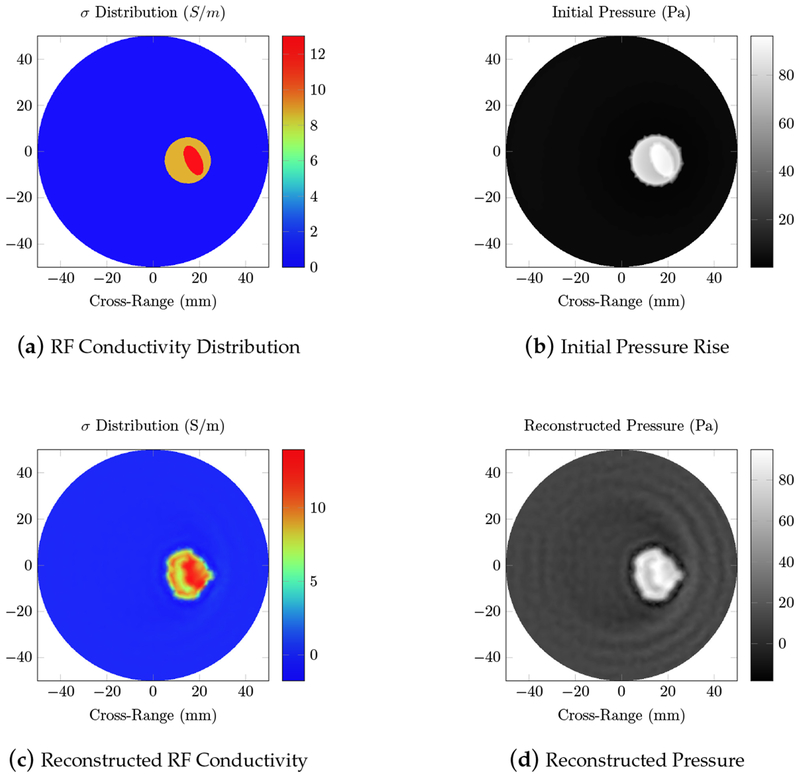
RF-induced acoustic computed tomography (RACT) simulations of the breast phantom using a 434 MHz RF source; (**a**) the true RF conductivity; (**b**) the initial pressure rise induced by the thermoacoustic effect; (**d**) the reconstructed pressure, calculated using the time-reversal algorithm; and (**c**) the reconstructed absorption, found by dividing the reconstructed pressure by the electric field intensity. The elliptical region with significantly larger conductivity is the tumor, surrounded by the glandular tissue.

The forward simulation of the electric field is able to directly detect the tumor and an estimation of its location. The conductivity has a much more significant effect on the wave, since the wavelength is larger than the region of interest. In the large wavelength regime, scattering due to dielectric contrast does not distort the electromagnetic wave as much as the substantial conductivity. While the approximate location of the tumor can be inferred from the electric field intensity, the specific shape and glandular tissue identification requires further processing, such as the RF acoustic simulation. Figure 9 shows the initial pressure and the resulting reconstructed pressure and conductivity maps of the breast phantom.

## 4. Discussion

### 4.1. Merits of ONELAB for Thermoacoustic Imaging

Using an open source platform, ONELAB, allowed a single interface to simulate hybrid thermoacoustic imaging, with the choice of optical, radio-frequency, or other sources to induce a pressure wave response. The components of ONELAB Gmsh (mesh) and GetDP (FEM solver) were applied for this purpose. Since GetDP only solves the user defined finite element equations, fine control over every aspect of the thermoacoustic simulation was possible. This is especially important when performing reconstruction, since, for instance, time-reversal can have drastically different performance depending on the amount of time steps and the duration per time step. The benefits of Gmsh include its standard mesh creation, with the ability to automatically interface with other standard mesh formats. This allows the user to write a wrapper around any given finite element mesh. Besides simulating expected results, this platform is also ideal for post-processing the results. All of the data from the simulated PACT and RACT experiments was in the same format, used the same finite element mesh, without the need for interpolation or data transformation. Using the data in such a complementary fashion demonstrated the benefits of ONELAB over more established simulation tools used for a single imaging modality.

### 4.2. Simulation Workflow for both PACT and RACT

We first tested our algorithms on a simple phantom that consisted of two absorbing regions of different shapes inside a homogeneous background. The second phantom simulated a human breast with a tumor. The same phantom mesh was used to define both optical and RF-acoustic properties of the tissue. The optical diffusion equation was solved to obtain the optical fluence maps of the phantoms (Figures 2 and 6), while the solution to Maxwell’s equations mapped the electric field distribution for these phantoms (Figures 4 and 8). Subsequently, maps of the initial pressure rises, due to electromagnetic absorption and the thermoacoustic effect, were generated using the optical fluence maps in PACT and electric field intensity in RACT. The initial pressure distributions of both PACT and RACT were then propagated using the same time-domain equations. Respective maps of the reconstructed pressure were generated using a time-reversal algorithm. Optical absorption and conductivity of the tissue phantoms were recovered by dividing the related reconstruction pressures by the optical fluence and electric field intensity distributions, respectively (Figures 3, 5, 7 and 9).

### 4.3. Analysis of PACT and RACT Results

Overall, our simulation results on two different tissue phantoms have shown that ONELAB can effectively simulate photoacoustic computed tomography (PACT) as well as RF-induced acoustic computed tomography (RACT). The optical fluence distribution (Figure 2) and electric field intensity (Figure 4) for the homogeneous phantom were significantly different, though the PACT/RACT reconstruction (Figures 3 and 5) is of similar accuracy. The relative error of the maximum absorption coefficient was 1.25% for the PACT case, and 8.70% in the RACT case. PACT for this phantom had a much lower relative error, since the contrast between absorber was much larger than the same difference in the RACT case. Imperfect reconstruction of the absorbers is due to error in reconstructing the initial pressure, instead of differences in the field intensity distribution. The relative shape of the absorbers, using either PACT or RACT, is easily identified from the reconstruction, with further processing only necessary for very precise characterizations. The successfully simulated results on a generic phantom gave us confidence to further validate our algorithms on a real tissue phantom mimicking the optical and RF properties of a human breast.

When the approximate optical and RF parameters corresponding to breast tissue are used (Figures 6 and 8), the optical and electric field intensities resemble each other, and have similar accuracy to the homogeneous phantom. The relative error of the maximum absorption coefficient was 23.2% for PACT and 6.92% for RACT. In the realistic tissue, RACT had more contrast, leading to less error in the reconstruction. The difference in field intensity between the homogeneous and breast phantom did not have a significant effect on the reconstruction accuracy. Both PACT and RACT are able to approximately reconstruct the tumor and the surrounding glandular tissue with no further processing. Even though the tumor had properties similar to the background, the PACT reconstruction was still able to identify the tumor surrounded by the glandular tissue. In RACT, the tumor is readily identified as being significantly different from the background.

In summary, our work demonstrated that ONELAB is a viable simulation platform for use in PACT and RACT, and is well suited for experiments that exploit both modalities. Reconstructed images of the phantoms provided both qualitative and quantitative information about the tissue optical and conductivity properties, including the size, shape, and location of the target regions. This work laid a foundation for future studies to develop and validate more robust multimodality reconstruction algorithms that will help improve quantitative accuracy.

## 5. Conclusions

This study has demonstrated the use of the tools Gmsh and GetDP, known together as ONELAB, as a single simulation platform for modeling both optical, as well as RF-induced, thermoacoustic computed tomography, i.e. PACT and RACT, respectively. To achieve PACT and RACT results, the propagation of optical, radio frequency, and acoustic waves were effectively modeled using solutions of the optical diffusion equation, Maxwell’s equations, and time-domain wave equations. We validated our PACT and RACT algorithms using two types of tissue mimicking phantoms: a homogeneous phantom consisting of two absorbing targets and a breast phantom consisting of a tumor, with pre-defined optical and RF properties. Our results demonstrated that the optical and RF absorption properties of the respective tissue phantoms were accurately reconstructed using the proposed dual-modality computed tomography simulations in ONELAB. The use of the ONELAB software package allows for accurate multimodal thermoacoustic modeling, in order to develop and validate more robust algorithms for functional imaging of the human body.

## Figures and Tables

**Table 1.** Optical properties (*μ_a_* the absorption coefficient, and $\mu_{s}^{\prime }$ the reduced scattering coefficient) of different breast tissue at 800 nm.

Tissue	*μ_a_* (mm^−1^)	$\mu_{s}^{\prime }\;({\mathrm{mm}}^{-1})$
Background	0.0005	1.5742
Glandular	0.0059	1.12
Tumor	0.0021	0.625

**Table 2.** Electrical properties (ε_*r*_ the relative permittivity, and *σ* the conductivity) of different breast tissue at 434 MHz.

Tissue	*ε_r_*	*σ* (S/m)
Background	5.51	0.0353
Glandular	61.3	8.86
Tumor	25.25	13.03

## Abbreviations

The following abbreviations are used in this manuscript:

ONELAB: Open Numerical Engineering Laboratory
GetDP: General Environment for the Treatment of Discrete Problems
RF: radio frequency
CT: computed tomography
PET: positron emission tomography
MRI: magnetic resonance imaging
UCT: ultrasound computed tomography
PACT: photoacoustic computed tomography
RACT: radio frequency acoustic computed tomography
TACT: thermoacoustic computed tomography
FEM: finite element method
FDM: finite difference method
PDE: partial differential equation
CAD: computer-aided design
IGES: initial graphics exchange specification
STL: stereolithography
DICOM: Digital Imaging and Communications in Medicine
NIR: near infrared
TM: transverse magnetic
PML: perfectly matched layer
